# Contrast-enhanced ultrasound microbubble uptake and abnormal plasma biomarkers are seen in patients with abdominal aortic aneurysms

**DOI:** 10.1016/j.jvssci.2025.100284

**Published:** 2025-03-08

**Authors:** Adham N. Abou Ali, Patrick Cherfan, Ashraf G. Taha, Michel S. Makaroun, Yingze Zhang, Xucai X. Chen, Flordeliza S. Villanueva, Rabih A. Chaer

**Affiliations:** a Division of Vascular Surgery, University of Pittsburgh Medical Center, Pittsburgh, PA; b Division of Vascular Surgery, Charleston Area Medical Center, Charleston, WV; c Department of Vascular and Endovascular Surgery, Assiut University Hospital, Assiut, Egypt; d Division of Pulmonary, Allergy and Critical Care Medicine, University of Pittsburgh Medical Center, Pittsburgh, PA; e Department of Medicine, University of Pittsburgh School of Medicine, Pittsburgh, PA

**Keywords:** Contrast enhanced ultrasound, Abdominal aortic aneurysms, Contrast ultrasound uptake

## Abstract

**Objective:**

Abdominal aortic aneurysm (AAA) growth is unpredictable. We hypothesize that contrast-enhanced ultrasound (CEUS) imaging and plasma inflammatory biomarkers (PIBs) may detect AAA wall inflammation.

**Methods:**

Patients with an AAA diameter ≥4 cm had CEUS and PIB testing at enrollment and every 6 months. Microbubble replenishment was analyzed via manually drawn regions of the aortic wall. Aneurysm growth, rupture, and repair were recorded. PIB testing was analyzed using biomarker panels. Independent and paired *t*-tests were used to detect differences in PIB levels. Logistic regression was used to study the association between PIBs, microbubble uptake, and AAA growth.

**Results:**

A total of 59 patients were enrolled (mean age, 68.8 ± 8.6 years; 13.6% female; 93.2% White). Mean AAA size on presentation was 41.6 ± 6.7 mm. Microbubble uptake was seen in 36 patients (61%). Patients with AAA had high baseline levels of Cystatin C and interferon-γ and low levels of macrophage migration inhibitory factor. Microbubble uptake was seen in 59% of patients with ≥5 mm AAA growth but was not predictive of growth on logistic regression.

**Conclusions:**

We have demonstrated that microbubble uptake with CEUS is seen in the aortic wall/intraluminal thrombus of patients with AAA. CEUS and PIBs could provide insight into aneurysm behavior in newly diagnosed AAA.

## Clinical Relevance

Microbubble contrast agents hold significant clinical relevance for the evaluation of aneurysms, offering an innovative approach to enhancing diagnostic imaging and improving patient outcomes. These agents, when used in ultrasound or other imaging modalities, allow for the detailed visualization of tissue characteristics within the aneurysm wall. By enhancing the contrast between the aneurysm and surrounding tissues, microbubbles provide a more precise assessment of wall integrity and vascularization by assessing the vasa vasorum density. This improved visualization can be crucial for identifying aneurysms that are at higher risk of rupture, enabling timely interventions such as surgical repair or endovascular procedures. Additionally, microbubble contrast can help in monitoring the effectiveness of treatments or interventions over time, offering a non-invasive, repeatable method for tracking aneurysm progression and endoleak detection. Ultimately, the use of microbubble contrast in aneurysm evaluation could lead to more personalized treatment strategies, reducing the risk of rupture and improving patient prognosis through better-informed decision-making.

Aneurysm size and growth rate have been the predominant determinants of abdominal aortic aneurysm (AAA) clinical evaluation and management. Unfortunately, they do not fully explain aneurysm behavior. Contrast-enhanced ultrasound (CEUS) is a noninvasive imaging modality that carries a particular advantage over conventional ultrasound techniques, such as the ability to detect slow flow in small vessels.^1^ Although CEUS imaging of the abdominal aorta after endovascular repair is utilized for the detection of endoleaks, its role in informing clinicians about aneurysm growth patterns is unknown.^1^

Data from the carotid literature shows that CEUS allows atherosclerotic plaque analysis by dynamic assessment of the vasa vasorum (VV).^2^ The presence of intraplaque vessels detected by CEUS was significantly associated with plaque hemorrhage, suggesting CEUS as a valuable tool for assessing the risk of cerebrovascular and cardiovascular events.^3^, ^4^, ^5^ Its role in AAAs is linked to the ability of CEUS to examine the VV of the aorta given the link between adventitial VV and aortic disease pathogenesis.^6^, ^7^, ^8^ An area of increased uptake in the aneurysm wall may indicate wall instability, which allows CEUS to provide additional information beyond the data gleaned from a contrasted computed tomography scan or a plain duplex ultrasound.

The role of plasma inflammatory biomarker (PIB) testing in AAA pathophysiology has been previously highlighted. The upregulation of C-reactive protein (CRP) and interleukin (IL)-6 in patients with AAAs suggests an activation of the inflammatory response system, which has become well-known in aneurysm physiology.^9^^,^^10^ Certain biomarkers such as CRP and other inflammatory cytokines were found to positively correlate with increasing aneurysm size and abdominal aneurysm peak wall stress.^11^

We hypothesize that CEUS imaging may detect AAA wall and intraluminal thrombus (ILT) inflammation and neovascularization with VV seen with microbubble uptake, which in conjunction with PIBs, may identify patients at risk for AAA growth/rupture. This will potentially allow for an individualized surveillance protocol of patients with AAA based on the biological activity of the aortic wall.

## Methods

The study protocol was approved by the Institutional Review Board of the University of Pittsburgh (STUDY19120107) and registered on clinicaltrials.gov (NCT02022436). The study was funded by the AGS GEMSTAR award from the Society of Vascular Surgery foundation in addition to the National Institutes of Health (NIH) through an R03 grant.

### Study design

The objective of this study was to study the metabolic processes within the aortic wall that are thought to explain aortic growth noninvasively. This was a single-center prospective evaluation of patients with an AAA diameter of at least 4.0 cm enrolled between December 2011 and December 2016, in the outpatient vascular clinic of the University of Pittsburgh Medical Center. Patients were followed up until October 2019. PIB testing was performed using biomarker panels (Meso scale discovery) and Bioplex 100 analyzer (Bio-rad).

Aneurysm diameter size was determined on duplex ultrasound. Patients were excluded if they had contraindications to the administration of Definity contrast, such as pulmonary hypertension, unstable cardiopulmonary conditions, known right-to-left cardiac shunting, and pregnancy. Patients underwent CEUS and serum PIB testing at enrollment and every 6 months up for 18 months or until eventual repair. PIB testing was also done 1 year after AAA repair.

### CEUS imaging protocol

**Fig 1:**
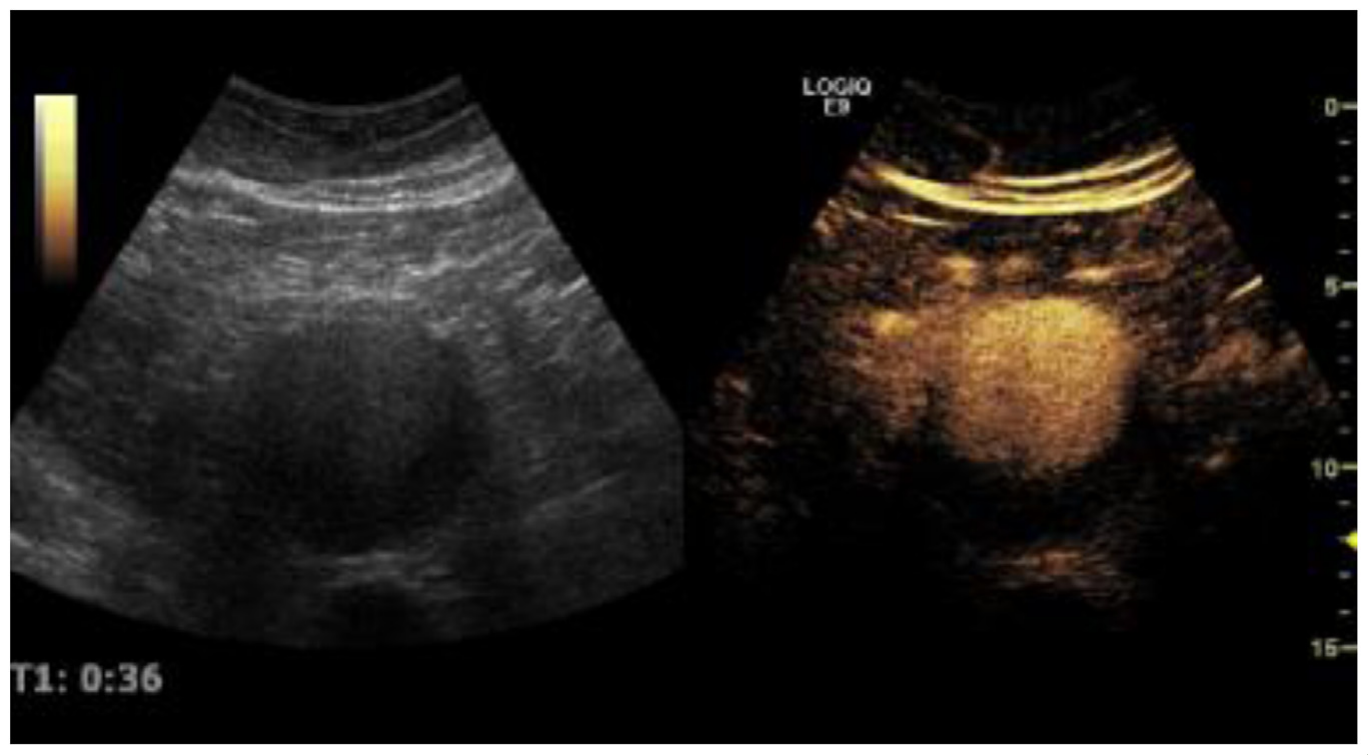
B-mode ultrasound of an abdominal aortic aneurysm (AAA) (*left*). Contrast-enhanced visualization of the AAA prior to microbubble disruption by ultrasound waves (right).

Patients were positioned on their back in the non-fasting state. An intravenous line was placed by the research staff at the time of the office visit. The abdominal aorta was imaged initially without contrast administration. Following non-contrasted duplex imaging, CEUS was performed via intravenous administration of the ultrasound contrast agent, Definity (Lantheus). One mL of the contrast agent was diluted in 9 mL of normal saline to achieve a concentration of approximately 10 microliters/kg. For each injection sequence, 0.5 mL of the diluted contrast was injected over 2 to 3 seconds during simultaneous burst replenishment real-time imaging. Two injection sequences were typically performed. Each CEUS evaluation took approximately 20 to 30 minutes to complete. Patient’s heart rate, blood pressure, and breathing were monitored during the examination and afterwards in the waiting area. The total monitoring time ranged between 30 and 90 minutes. Microbubble replenishment was analyzed using ImageJ (NIH) via manually drawn regions of the aortic wall and ILT (Fig 1).

Ultrasound evaluation was performed using the Logiq E9 US system (GE Healthcare) with the standard curved array abdominal transducer. The contrast settings on the ultrasound system were set to a low mechanical index of 0.07 and a dynamic range of 50, with the focal zone placed underneath the posterior aortic wall.

**Fig 2:**
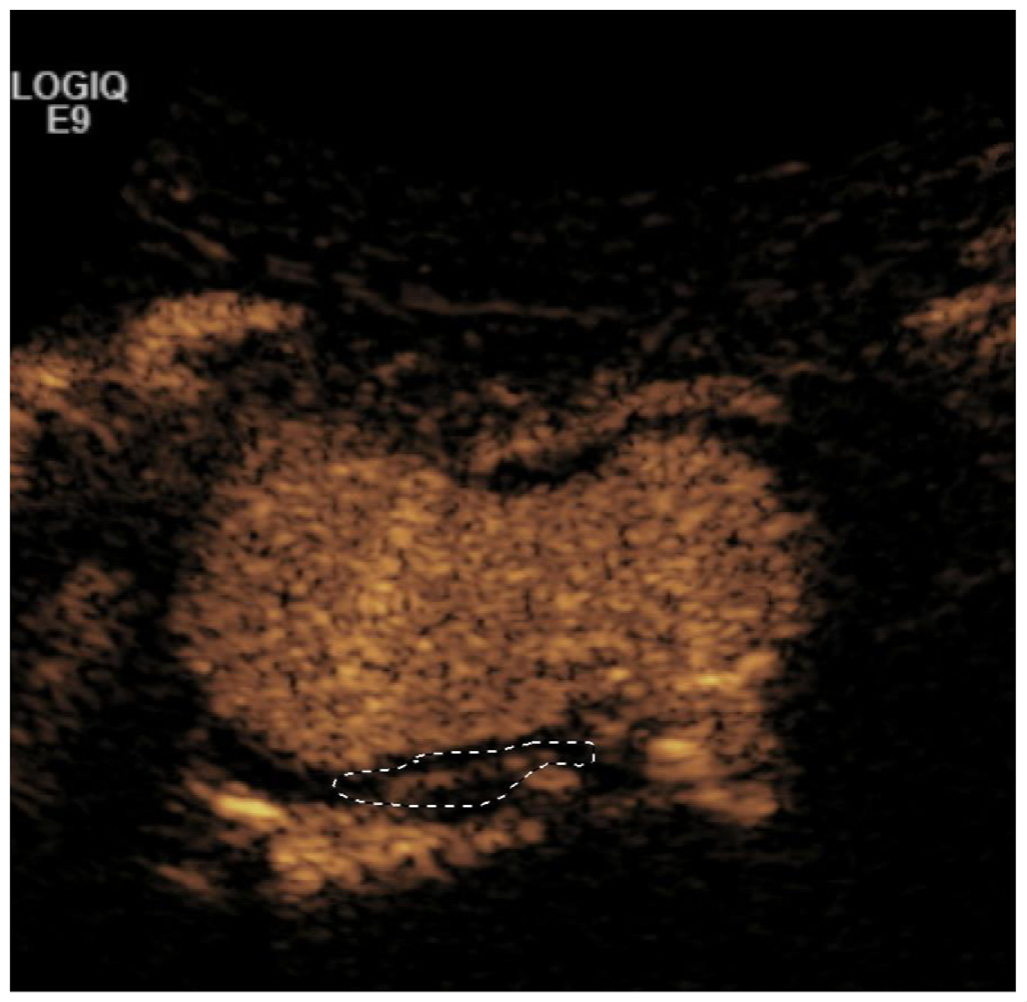
Microbubble uptake seen in the posterior aortic aneurysm wall.

Microbubble uptake was determined by the ultrasound technologist, and the location was recorded on a study sheet. The same ultrasound technologist performed the microbubble study throughout the entire period. A workflow sheet was created at the index visit with annotation of the area of uptake to facilitate follow-up imaging while minimizing intraobserver variability. Uptake was then separately recorded by two of the investigators (R.C., P.C.). Both investigators met and adjudicated each region of interest with its corresponding contrast uptake behavior to fully maximize interobserver reliability. Consensus agreement was reached for every patient. Although the extent of uptake can be determined using video intensity analysis, it was not pursued in this study. Care was taken to correlate with color flow duplex ultrasound to avoid lumbar and mesenteric vessels as well as artifact. The visualization of the microbubbles within the AAA wall was defined as contrast uptake at the index visit. This was determined by consensus agreement between the ultrasound sonographer and two investigators (R.A.C. and P.C.) for every patient. Microbubble uptake and replenishment was analyzed using ImageJ (NIH) via manually drawn regions of the aortic wall and ILT. (Fig 2).^12^ Aneurysm growth, rupture, and repair were recorded during the follow-up period.

### Biomarker testing

Blood samples were collected from patients at each visit and 1 year following AAA repair. Blood samples were processed, and plasma samples were stored at −80 ^o^C immediately for future biomarker analysis. The biomarkers studied were chosen based on published literature suggesting a potential association with aneurysm disease while balancing resource optimization.^13^ The plasma levels of interferon γ (IFN**-**γ), IL10, IL6, IL8, and tumor necrosis factor-α (TNF-α) were analyzed using the Proinflammatory Panel 1 (MSD); CRP, intercellular adhesion molecule (ICAM1), serum amyloid A (SAA), and vascular cell-adhesion molecule (VCAM1) using Vascular Injury Panel 2 (MSD); macrophage migration inhibitory factor (MIF) using the Human MIF kit (MSD); osteoprotegerin (OPGN) using the Human Bone Panel 1 (MSD); cystatin C, neutrophil gelatinase-associated lipocalin (NGAL), and uromodulin (UMOD) using the Kidney Injury Panel 5 (MSD); and galectin 3 using an enzyme-linked immunosorbent assay kit (US IVD BGM) according to the instructions from the manufacturers.

### Statistical analysis

Independent *t*-tests were used to detect differences in PIB levels between patients with AAA with and without contrast uptake. Paired *t*-tests compared PIB levels in patients (baseline vs follow-up). Logistic regression was used to study the association between PIBs, microbubble uptake, and AAA growth. Statistical significance was deemed at *P*-value < .05. Data analysis was performed using Stata (16.1, StataCorp LLC).

## Results

### Baseline demographics

A total of 59 patients with AAAs were enrolled (mean age, 68.8 ± 8.6 years; 13.6% female; 93.2% White). Of the patients, 86.2% were current or former smokers, and 84.5% were on statin therapy. Patients were tested at follow-up timepoints: 22 patients underwent repair after the first visit, and 14 patients gradually progressed requiring repair at a later point. Mean follow-up of patients was 59.0 ± 43.5 months. There were no baseline demographic or comorbidity differences between patients with aneurysm who had microbubble uptake compared with patients who did not (Table I). Seventeen patients (28.8%) died throughout the follow-up period, including seven patients who had undergone prior aneurysm repair; none of the deaths were related to aneurysm rupture.

**Table I:** Baseline demographics and comorbidities stratified by microbubble uptake

	Microbubble uptake		*P*-value
	Yes (n = 36)	No (n = 23)	
Age, years	68.6 ± 8.6	69.1 ± 9.0	.84
Male gender	33 (91.7)	18 (78.2)	.14
White race	33 (91.7)	22 (95.7)	.55
Coronary artery disease	19 (54.3)	14 (60.1)	.62
Diabetes	8 (22.9)	6 (26.1)	.78
Statin use	29 (82.9)	20 (87.0)	.32
Average AAA diameter, mm	42.0 ± 7.1	41.2 ± 6.2	.68
AAA repair	20 (55.6)	16 (69.5)	.28
Aneurysm growth rate, mm/year	3.0 ± 3.1	3.2 ± 3.1	.78

*AAA,* Abdominal aortic aneurysm.

Data are presented as number (%) or mean ± standard deviation.

### AAA characteristics

Mean AAA diameter on enrollment was 41.7 ± 6.7 mm. Microbubble uptake was seen in 36 patients (61%). Mean AAA growth rate was 3.0 ± 3.1 mm/year (range, 0-15.7 mm/year). There was no statistically significant difference between growth rates/year between patients with vs without contrast uptake (Table I). Patients with no uptake had a higher proportion of requiring aneurysm repair compared with patients with uptake; however, this did not reach statistical significance (69.5% vs 55.6%; *P* = .282). Microbubble uptake was seen in 59% of patients with ≥5 mm AAA annual growth rate, but this was not predictive of growth on logistic regression. No adverse events related to the administration of the ultrasound contrast agent were reported.

### Biomarker analysis

#### Biomarker levels and aneurysm growth

Serum levels of biomarkers were measured at enrollment, at every follow-up visit and at 1 year after repair. The mean baseline concentration of biomarkers in patients with AAAs is reported in Table II.

**Table II:** Mean baseline levels of plasma inflammatory biomarkers (*PIBs*) among patients with aneurysms

PIBs	Patients with AAA (N = 59)
OPGN, pg/mL	226 ± 83
Galectin 3, ng/mL	18.5 ± 6.6
Cystatin C, ng/mL	1585 ± 832
NGAL, ng/mL	190 ± 107
UMOD, ng/mL	79.1 ± 55.5
CRP, ng/mL	7090 ± 13202
MIF, pg/mL	8905 ± 13764
ICAM1, ng/mL	417 ± 111
IFN-y, pg/mL	4.2 ± 3.4
IL10, pg/mL	0.2 ± 0.2
IL6, pg/mL	1.4 ± 1.1
IL8, pg/mL	7.2 ± 5.5
TNF-α, pg/mL	3.0 ± 1.7
SAA, ng/mL	12865 ± 38272
VCAM1, ng/mL	539 ± 340

*AAA,* Abdominal aortic aneurysm; *CRP,* C-reactive protein; *ICAM1,* intercellular adhesion molecule; *IFN-γ,* interferon γ; *IL10,* interleukin 10; *IL6,* interleukin 6; *IL8,* interleukin 8; *MIF,* macrophage migration inhibitory factor; *NGAL,* neutrophil gelatinase-associated lipocalin; *OPGH,* osteoprotegerin; *SAA,* serum amyloid A; *TNF-α,* tumor necrosis factor-α; *UMOD,* uromodulin; *VCAM1,* vascular cell-adhesion molecule.

In patients with aneurysm growth and sequential biomarker testing (n = 37), OPGN levels increased from 227.4 ± 84.6 pg/mL at the index visit to 257.4 ± 97.1pg/mL at the time of first follow-up (*P* = .004). Mean aneurysm size remained about the same from 46.4 ± 5.8 mm at baseline to 46.5 ± 4.3 mm at the time of the first follow-up; this was not statistically significant. Conversely, UMOD levels decreased from 86.7 ng/mL to 68.4 ng/mL (*P* = .002). No other biomarker differences were noted between the two time points. Similarly, there were no other differences in biomarker levels at different future time points.

There was a statistically significant positive correlation between baseline OPGN levels and mortality while adjusting to baseline comorbidities (B = 0.02 [0.0004-0.03] [*P* = .014]).

#### Biomarker levels in patients with microbubble uptake

There was no difference in the mean biomarker levels at baseline between patients with microbubble uptake and those without (*P* > .05). Comparing the change in biomarker levels at different follow-up time points between patients with and without microbubble uptake, OPGN, MIF, and cystatin C level changes at first follow-up were significantly higher in patients with microbubble uptake compared with level changes in patients without microbubble uptake (*P* < .05) (Table III).

**Table III:** The change in plasma inflammatory biomarkers (*PIBs*) stratified by microbubble uptake

PIBs	Microbubble uptake					
	Yes (n = 20)			No (n = 17)		
	Baseline	Follow-up	*P*-value	Baseline	Follow-up	*P*-value
OPGN, pg/mL	218 ± 77	250 ± 101	**.02**	239 ± 94	266 ± 95	.10
Galectin 3, ng/mL	17.6 ± 5.6	18.6 ± 6.6	.25	21.3 ± 8.8	23.4 ± 17.4	.45
Cystatin C, ng/mL	1449 ± 735	1657 ± 1080	.09	1960 ± 831	1684 ± 750	.11
NGAL, ng/mL	183 ± 140	189 ± 139	.65	230 ± 87	219 ± 108	.53
UMOD, ng/mL	81.3 ± 61.4	64.9 ± 59.4	**.003**	93.0 ± 68.4	72.4 ± 57.3	**.02**
CRP, ng/mL	4576 ± 4700	4582 ± 3832	.99	9442 ± 20667	11604 ± 33460	.53
MIF, pg/mL	8850 ± 19349	12370 ± 24074	.03	13090 ± 13365	8162 ± 8353	.10
ICAM1, ng/mL	424 ± 121	427 ± 183	.92	450 ± 133	403 ± 92	**.01**
IFN-y, pg/mL	4.8 ± 3.2	5.8 ± 5.6	.29	3.9 ± 2.9	4.2 ± 2.7	.58
IL10, pg/mL	0.2 ± 0.1	0.2 ± 0.2	.75	0.2 ± 0.1	0.5 ± 1.3	.34
IL6, pg/mL	1.4 ± 1.0	1.7 ± 1.3	.12	1.6 ± 1.5	7.6 ± 24.1	.31
IL8, pg/mL	6.1 ± 2.3	8.2 ± 6.7	.100	7.3 ± 2.7	6.9 ± 2.7	.235
TNF-a, pg/mL	2.6 ± 0.8	2.9 ± 1.0	.088	3.9 ± 2.8	3.5 ± 2.3	.143
SAA, ng/mL	10534 ± 22080	7775 ± 10907	.360	23444 ± 66087	20065 ± 65536	.212
VCAM1, ng/mL	499 ± 242	507 ± 242	.795	655 ± 547	625 ± 487	.160

*AAA,* Abdominal aortic aneurysm; *CRP,* C-reactive protein; *ICAM1,* intercellular adhesion molecule; *IFN-γ,* interferon γ; *IL10,* interleukin 10; *IL6,* interleukin 6; *IL8,* interleukin 8; *MIF,* macrophage migration inhibitory factor; *NGAL,* neutrophil gelatinase-associated lipocalin; *OPGH,* osteoprotegerin; *SAA,* serum amyloid A; *TNF-α,* tumor necrosis factor-α; *UMOD,* uromodulin; *VCAM1,* vascular cell-adhesion molecule.

Boldface *P* values indicate statistical significance.

Similarly, the change in ICAM-1 levels significantly decreased in patients without microbubble uptake from 449.5 ± 132.5 ng/mL to 403.1 ± 92.1 ng/mL (*P* = .002) while staying the same in patients with uptake (*P* > .05). There was no statistically significant difference in biomarker level changes at different follow-up time points between patients with vs without microbubble uptake.

## Discussion

The current treatment paradigm for AAAs has been centered on aneurysm size and growth rate, given the association between these two factors and subsequent aneurysm rupture. Serial ultrasound or computed tomography scan imaging is recommended by societal guidelines based on aneurysm size. Surveillance imaging is recommended every 12 months for AAAs with a diameter between 4.0 and 4.9 cm and every 6 months for AAAs with a diameter between 5.0 and 5.4 cm.^13^ However, aneurysm size does not always reflect the aneurysm behavior. Autopsy studies have demonstrated that 13% of patients with AAAs smaller than 5.0 cm suffered a rupture, whereas 40% of those with AAAs between 7 and 10 cm did not suffer a rupture.^14^ A contemporary analysis of an AAA registry revealed that the annual rupture rates of large AAAs were “lower than previously reported.”^15^

AAA growth is highly variable not only between patients but also over time. It can be characterized by periods of growth alternating with periods of non-growth. These variable AAA growth patterns may reflect structural changes within the aneurysm wall, where tissue remodeling is associated with progressive degeneration of the aortic wall.^16^ This unpredictability suggests that there is more to AAA than aneurysm size. Computational fluid dynamic analysis of aortic aneurysms has shown that not all areas of the aneurysm are subject to the same amount of wall shear stress.^17^ It may follow that different areas of the same aneurysm can have variations in VV density and intraplaque neovascularization. The proposed eventual objective of analyzing VV density and plaque neovascularization in the aneurysm wall is to allow for a personalized approach for aneurysm treatment and surveillance. However, further research is needed to determine whether or not CEUS can play that role in aneurysm evaluation.

The involvement of adventitial VV takes place early in the atherosclerosis process even prior to intimal medial thickness.^18^ The adventitial network later extends further inward and contributes to intraplaque neovascularization. Neovascularization in arterial walls appears to be a histopathologic marker of wall ischemia, regional inflammation, occlusive vascular episodes, and aneurysmal degeneration. This neovascularization as represented by the VV is increased compared with normal tissue in atherosclerotic and aneurysmal lesions. Microbubble contrast accumulates in these hypervascularized areas, allowing for their detection via ultrasound.^5^ CEUS can also detect adventitial VV, which, in carotid lesions, correlated with more cardiovascular disease. Intraplaque neovascularization detected with CEUS correlated with histologic specimens from carotid lesions after endarterectomies. Similarly, VV neovascularization has been identified in high-risk aortic plaque features.^16^^,^^18^ The detection and quantification of neovascularization within the wall of AAA via CEUS offers promise for further risk stratification and identification of patients who are more susceptible to increased growth and rupture.

The study reveals that microbubble uptake was seen in 61% of patients. This is comparable to CEUS examinations of 147 carotid artery lesions among asymptomatic patients, which showed intraplaque uptake in 54% of patients.^5^ In their analysis, uptake was associated with increased odds of cardiovascular events. Microbubble uptake as a surrogate of VV density suggested a vulnerable lesion and a predictor of cardiovascular events.^12^ The relatively high proportion of uptake in the cohort was not associated with the need for repair. The data did not support the hypothesis that VV uptake was associated with an increased risk for operative repair. This could be due to the small sample size, limited longitudinal follow-up, and the fact that microbubble uptake was determined in patients who were expected to get their aneurysm repair soon.

The impetus for using serum circulating biomarkers to risk stratify aneurysms is to provide an individualized patient surveillance strategy while not solely relying on aneurysm diameter.^19^ Similar to what has been reported in the literature, the study suggests that PIBs are upregulated in patients with AAA. The selection of the inflammatory biomarkers in this study was based on published literature and on attempts to optimize resource utilization. Local tissue hypoxia in the aneurysm wall stimulates the production of growth factors, cytokines, and matrix metalloproteinases. These inflammatory mediators, in turn, promote extracellular matrix degradation that results in gradual weakening of the aortic wall and further contributes to progressive aortic dilatation.^20^ Osteoprotegerin and macrophage migration inhibitory factors were biomarkers shown to have increased expression in patients with AAAs. OPGN activates matrix metalloproteinases that in turn trigger the destruction of the aortic wall; the levels of this glycoprotein cytokine receptor have been correlated with larger AAA diameter.^21^^,^^22^ The analysis suggests that those biomarkers may be more pronounced in patients with aneurysm growth, and specifically those with microbubble uptake. Compared with patients without microbubble uptake in which ICAM-1 levels decreased, ICAM-1 levels remained the same in patients with uptake, suggesting a persistent inflammatory response. UMOD levels were noted to be significantly lower in patients who experienced aneurysm growth. This is consistent with the literature’s inverse correlation between UMOD and other inflammatory biomarkers such as CRP.^23^ In the analysis, patients with AAA had a high concentration of OPGN and TNF-α, which have been associated with aneurysm progression, given their role in the inflammatory process.^24^ CRP and SAA, like other transmembrane enzymes, have also been positively correlated with increasing aneurysm size.^9^, ^10^, ^11^ However, the clinical utility of biomarkers in patient risk stratification remains uncertain. New data has also emerged to suggest that inflammatory biomarkers can also serve as adjuncts in monitoring patients post-endovascular aortic repair to determine which patients might develop endoleaks.^25^

The small sample size of the study prevents robust and definitive conclusions. The lack of a significant correlation between PIBs and microbubble uptake is likely attributed to the small sample size. In addition, CEUS imaging of the aortic wall had not been previously performed at our institution, so there was an initial learning curve before the protocol was fully established. Intraobserver variability was not ascertained; however, using the same technologist who recorded the uptake locations helped minimize the variability. Other limitations are inherent to ultrasound imaging in general, with image quality being dependent on patient body habitus and overlying bowel gas. However, care was taken to have a dedicated ultrasonographer for the study, and the current experience reflects the research setting where an experienced sonographer and interpreting physicians were the primary sources of data ascertainment. Specific challenges to CEUS imaging of the AAA include the relatively large area to image from the renal arteries to the aortic bifurcation (compared with a carotid plaque, for example). This makes serial ultrasound evaluations of exact locations difficult and keeping an uptake log on a diagram of the AAA was critical for follow-up. Quantification of VV microbubble uptake was the main challenge in this study and other studies evaluating the role of CEUS. However, with an established protocol for AAA CEUS imaging, microbubble uptake was demonstrated in the aortic wall in a reproducible fashion using serial imaging.

## Conclusions

Microbubble uptake with CEUS is seen in the aortic wall and/or ILT of patients with AAAs. CEUS and PIBs could provide insight into aneurysm behavior in newly diagnosed AAAs, which sets the stage for predictive algorithms for AAA growth. Longitudinal studies using a larger sample size and longer follow-up are ultimately required to ensure the reproducibility and reliability of contrast-enhanced microbubble imaging for aortic aneurysm imaging. The challenge of utilizing biomarkers in clinical AAA evaluation and risk stratification remains significant in light of the inconsistent results. Individualized aortic surveillance strategies combining anatomic, biochemical, and radiologic parameters remain the target for future research.

## Funding

This study was funded by the AGS GEMSTAR award from the Society of Vascular Surgery Foundation in addition to the 10.13039/100000002National Institutes of Health through an R03 grant.

## Disclosures

None.
